# Strong selection and high mutation supply characterize experimental *Chlorovirus* evolution

**DOI:** 10.1093/ve/veac003

**Published:** 2022-01-25

**Authors:** Cas Retel, Vienna Kowallik, Lutz Becks, Philine G D Feulner

**Affiliations:** Department of Fish Ecology and Evolution, Center for Ecology, Evolution and Bio-geochemistry, EAWAG, Swiss Federal Institute of Aquatic Science and Technology, Seestrasse 79, Kastanienbaum 6047, Switzerland; Division of Aquatic Ecology, Institute of Ecology and Evolution, University of Bern, Baltzerstrasse 6, Bern 3012, Switzerland; Department of Evolutionary Ecology, Community Dynamics Group, Max Planck Institute for Evolutionary Biology, August-Thienemann-Str. 2, Plön 24306, Germany; Aquatic Ecology and Evolution, Limnological Institute University Konstanz, Mainaustraße 252, Konstanz / Egg 78464, Germany; Department of Fish Ecology and Evolution, Center for Ecology, Evolution and Bio-geochemistry, EAWAG, Swiss Federal Institute of Aquatic Science and Technology, Seestrasse 79, Kastanienbaum 6047, Switzerland; Division of Aquatic Ecology, Institute of Ecology and Evolution, University of Bern, Baltzerstrasse 6, Bern 3012, Switzerland; Department of Evolutionary Ecology, Community Dynamics Group, Max Planck Institute for Evolutionary Biology, August-Thienemann-Str. 2, Plön 24306, Germany

**Keywords:** virus evolution, genomics, repeatable genomic change, predicted phenotypic effect, Chlorovirus PBCV-1

## Abstract

Characterizing how viruses evolve expands our understanding of the underlying fundamental processes, such as mutation, selection and drift. One group of viruses whose evolution has not yet been extensively studied is the *Phycodnaviridae*, a globally abundant family of aquatic large double-stranded (ds) DNA viruses. Here we studied the evolutionary change of *Paramecium bursaria* chlorella virus 1 during experimental coevolution with its algal host. We used pooled genome sequencing of six independently evolved populations to characterize genomic change over five time points. Across six experimental replicates involving either strong or weak demographic fluctuations, we found single nucleotide polymorphisms (SNPs) at sixty-seven sites. The occurrence of genetic variants was highly repeatable, with just two of the SNPs found in only a single experimental replicate. Three genes *A122/123R, A140/145R* and *A540L* showed an excess of variable sites, providing new information about potential targets of selection during *Chlorella*–Chlorovirus coevolution. Our data indicated that the studied populations were not mutation-limited and experienced strong positive selection. Our investigation highlighted relevant processes governing the evolution of aquatic large dsDNA viruses, which ultimately contributes to a better understanding of the functioning of natural aquatic ecosystems.

## 1. Introduction

Viruses are found in large numbers in virtually any natural habitat. They can have enormous impacts on human society (World Health Organization 2021), can function as important ecological agents (Proctor and Fuhrman 1990; Suttle 2007; Rodriguez-Valera et al. 2009; Nelson et al. 2021) and play a large role in biogeochemical nutrient cycling (Bouvier and Del Giorgio 2007; Suttle 2007; Weitz and Wilhelm 2012). The ongoing discovery of new families of viruses (Yutin et al. 2009; Claverie and Abergel 2018; Krupovic et al. 2018; Pérez-Losada et al. 2020) repeatedly showed that some viruses have larger genomes, more complex particle structures and more sophisticated replication cycles than previously thought (Yutin et al. 2009; Warwick-Dugdale et al. 2019; Luo et al. 2020; Correa et al. 2021). Despite their important roles it is still unknown how some of these families of large viruses are able to adapt to changing environmental conditions (Short 2012; Claverie and Abergel 2013; Koonin and Yutin 2018). Understanding the molecular mechanisms underlying their evolution will help us elucidate their specific ecological role and is ultimately necessary to understand the functioning of complex natural ecosystems.

Viruses combine a distinct set of characteristics which are predicted to favor rapid adaptation. Their population sizes are generally large, with marine virus densities for example globally fluctuating around an average of 10^6^ particles ml^−1^ (Wommack and Colwell 2000; Wommack et al. 2015). They are also fast replicators, with e.g. *Paramecium bursaria* chlorella virus being able to complete a full cycle from initial infection to the release of new particles within 6 h (Van Etten et al. 1983) and *Mimivirus* needing up to 12 h (Yaakov et al. 2019). Estimates of genomic mutation rates for double-stranded (ds) DNA viruses are also orders of magnitude higher than those of pro- and eukaryotes, with 10^−6^ to 10^−8^ single nucleotide mutations per position per replication event (Sanjuán et al. 2010; Peck and Lauring 2018). Indeed, as a consequence of high population sizes, short generation times and high mutation rates, investigations into dsDNA viral genetic diversity often revealed evidence for recent rapid adaptation (e.g. Clavel and Hance 2004; Renzette et al. 2013).

However, there are other components of viral biology that might constrain their adaptation (Horas, Theodosiou, and Becks 2018). Viral abundances in freshwater ecosystems can seasonally fluctuate by several orders of magnitude (Yoshida et al. 2008) and between-host transmission events are often associated with strong population size bottlenecks. Viruses have high burst sizes (the number of newly synthesized particles produced per viral replication event), e.g. up to 350 in large dsDNA chlorella viruses (Van Etten et al. 1983) or up to 1,000 in other dsDNA viruses (Brussaard and Martínez 2008). Non-constant demographic histories and high variation in offspring numbers both increase the influence of stochastic forces during evolution, which decreases the genetic variation available for selection to act upon in the next generation (Gillespie 1998; Eldon and Wakeley 2006). Because viral genomes are densely packed with protein-coding regions, background selection (genetic diversity loss at non-deleterious loci; Charlesworth, Morgan, and Charlesworth 1993) can be comparatively strong, and this process can continuously remove genetic variation from viral populations, slowing down their adaptive evolution (Charlesworth, Morgan, and Charlesworth 1993; Cvijović, Good, and Desai 2018). Population size bottlenecks, unbalanced reproductive success and strong background selection all pose constraints to rates of viral adaptation (Irwin et al. 2016; Sanjuán 2018). Empirical information about how they interact with high population sizes, short generation times and high mutation rates to produce viral evolutionary change is still limited (but see Renzette et al. 2013; Pennings, Kryazhimskiy, and Wakeley 2014).

Chloroviruses are ubiquitous in temperate aquatic habitats around the world (Yamada, Onimatsu, and Van Etten 2006; Van Etten, Agarkova, and Dunigan 2020). They lyse their host to reproduce, and their seasonal abundance can fluctuate by three orders of magnitude, all of which suggest that they play an important ecological role in aquatic communities (Van Etten 2003; Yamada, Onimatsu, and Van Etten 2006). The type specimen for the genus is the strain *P. bursaria* chlorella virus 1 (PBCV-1). PBCV-1 infects the unicellular photosynthetic green alga species *Chlorella variabilis* NC64A, which is an endosymbiont of the protist *P. bursaria* (Karakashian, Karakashian, and Rudzinska 1968; Hoshina, Iwataki, and Imamura 2010). The specific host receptor molecule that PBCV-1 attaches to is unknown, but it is densely and evenly distributed across the outer cell surface (Meints et al. 1988). Quickly after attachment, the algal cell membrane is depolarized, which starts a cascade of events involving fusion of the viral and algal cell membranes that enables injection of viral DNA into the host (Agarkova et al. 2008; Romani et al. 2013). Because PBCV-1 prevents other viruses from entering the host cell after depolarization, genetic recombination between particles is very uncommon (Greiner et al. 2009). PBCV-1 is a globally ubiquitous large dsDNA virus of ecological relevance, and molecular details of the interaction with its host have partly but not completely been unraveled.

PBCV-1 has a genome size of 330 kb (Dunigan et al. 2012; Jeanniard et al. 2013) and encodes for 148 proteins, of which 106 have no known function or orthologs outside of large dsDNA viruses (Dunigan et al. 2012). Many proteins with functional orthologs have a putative function in carbohydrate manipulation (Van Etten et al. 2017). For the genes without functional orthologs outside the family of large dsDNA viruses, we rely on other evidence to reveal their putative functions: proteome analysis discloses which proteins are virion-associated (Dunigan et al. 2012), microarray and mRNA expression analyses reveal if a protein is expressed before or after viral DNA synthesis begins during infection (Yanai-Balser et al. 2010; Blanc et al. 2014), and a gene’s phylogenomic context suggests how essential it is (Jeanniard et al. 2013; Seitzer et al. 2018).

In summary, understanding how viruses evolve can help us elucidate their ecological role in natural ecosystems, as well as advance our understanding of the appearance and spread of pathogens (Geoghegan and Holmes 2018; Retel et al. 2019b). Here we investigated the genomic change in large dsDNA Chlorovirus populations during experimental coevolution with their algal host (Frickel, Sieber, and Becks 2016; Retel et al. 2019a). We were able to replicate the evolutionary process in a controlled chemostat environment, describe the genome-wide patterns of molecular evolution in this species and identify genes putatively involved in the coevolution with its host *C. variabilis*. Overall, the observed patterns of genomic variation indicated that mutation supply was high and that positive selection played an important role shaping Chlorovirus genomic change.

## 2. Materials and methods

### 2.1 Experimental system and design

**Figure 1.**
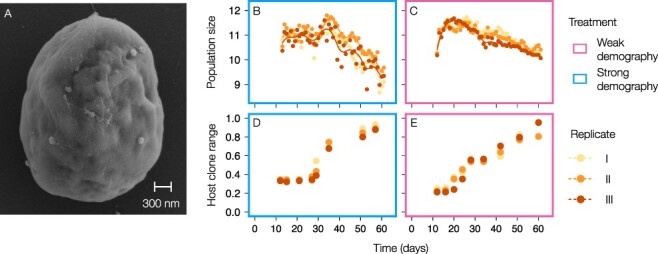
Chlorovirus PBCV-1 and its interaction with the host. A) Electron microscopy picture of a C. variabilis NC64A cell and several PBCV-1 particles on its outside. The icosahedral shape of the capsid is visible for some of the virus particles. B, C) Census population size dynamics of PBCV-1 during experimental coevolution including strong population size fluctuations (B) and under increased chemostat dilution rate which reduced population size fluctuations (C). Dots correspond to daily flow cytometric counts on the log_10_ scale, and the line is a smoothed average through them. D, E) Phenotypic change of PBCV-1 under strong population size fluctuations (D) and when population size fluctuations were reduced (E). Host clone range is the proportion of host individuals the virus population was able to infect, based on 80 (D) or 90 (E) phenotypic assays per population. The x-axis gives the time since the start of the experiment in days (virus was added at Day 12).

Six replicate Chlorovirus PBCV-1 populations were coevolved with *C. variabilis* NC64A hosts in a chemostat continuous culture setup (Frickel, Sieber, and Becks 2016; Retel et al. 2019a). The host strain *C. variabilis* NC64A was kindly provided by James Van Etten. The chemostats contained 800 ml of modified BBM (Bold’s basal medium, but nitrate was replaced by ammonium chloride), which was continuously resupplied at a fixed dilution rate. We maintained the chemostats under continuous light at 20°C and ensured the populations remained well mixed by uninterrupted stirring. An electron microscopy picture (Fig. 1A) shows a host cell with several virus particles on the outer surface of the host cell, and the characteristic icosahedral shape of PBCV-1 is clearly visible for some of them.

We inoculated the experiments with isogenic host populations, which were allowed to grow exponentially in the absence of virus for 12 days after inoculation (equaling approximately twelve host generations). Isogenic virus was added to all six replicates on the twelfth day. In three of the six replicates, we kept chemostat dilution rate at 0.1 day^−1^. Previous work showed that under these conditions, reciprocal antagonistic selection caused an arms race between the two species, characterized by strong population size fluctuations, directional increases in both host resistance and virus host range (Frickel, Sieber, and Becks 2016) and genomic evidence for adaptive sweeps in both populations (Retel et al. 2019a) (Strong Demography treatment, Fig. 1B, D). In the other three chemostats, we increased chemostat dilution rate to 0.3 day^−1^ to prevent the populations from undergoing strong population size fluctuations (Weak Demography treatment, Fig. 1C, E) (Becks et al. 2005). This study is part of a larger investigation and the data from the three ‘Strong Demography’ replicates were previously published to demonstrate how the interplay between selection and demography shapes genomic diversity during coevolution (Retel et al. 2019a). Here we expand by adding three replicates (Weak Demography treatment) and investigating the molecular evolution of the Chlorovirus populations in more detail.

In order to document population size changes over time, we took daily samples, which we fixed with 1 per cent glutaraldehyde, flash froze in liquid nitrogen and stored at −80°C. We used a flow cytometer (FACSCalibur, Becton Dickinson, San Jose, California) to quantify the density of virus particles (Brussaard 2004; Frickel, Sieber, and Becks 2016).

We assessed the host clone range of the virus populations at regular time intervals, following Frickel, Sieber, and Becks (2016) and Retel et al. (2019a). To determine whether a virus population was able to infect a host clone, we compared the host growth rates in the presence versus absence of the virus. We started these growth assays at an algal density with an optical density (OD) of 0.045 (Tecan, Infinite M200PRO, 680 Männedorf, Switzerland) and added virus to an initial MOI (Multiplicity Of Infection; here calculated as the ratio of virus to host particles) of < 0.01. We calculated host growth rates based on OD measurements at time point 0 and after 72 h. If the confidence intervals (mean ± 2 SD, calculated based on four technical replicates) of growth rates in the presence versus absence of the virus did not overlap, the virus was able to effectively kill the concerning host clone, and we classified it as infective. Assays with ancestral host clones served as positive controls for viral infectivity. Specifically, we tested virus population from eight time points in the ‘Strong Demography’ replicates and for nine time points in the ‘Weak Demography’ replicates. We randomly collected ten algal clones from the same set of time points from previously plated populations and regrew them in liquid BBM. We tested all virus populations against every clone from the replicate from which it was isolated (i.e. per Weak Demography replicate, nine virus populations were tested to see if they could infect 90 host clones, leading to 810 growth assays carried out in quadruplicate). Host clone range was finally calculated as the average number of host clones (from all time points) a virus could infect.

To look for differences in host clone range dynamics over time between the two experimental treatments, we ran a linear model in R (R Core Team 2008). We regressed host clone range over time in days, including an interaction term between time and treatment (which would capture a difference in host clone range increase between treatments).

To create the electron microscopy picture (Fig. 1A), exponentially growing algal cells were mixed with concentrated virus at an MOI of 10 and incubated on a shaker for 15 min (5,000 rpm). We transferred the cell suspension to multiwell plates containing a poly lysin coated cover slip and fixed the cells with 2 per cent glutaraldehyde on ice for 60 min. We carried out three washing steps with BBM on ice for 5 min each. Next, the cells were treated with 1 ml of 1 per cent OsO_4_ solution at 4°C for 60 min in the dark, followed by another three washing steps with BBM on ice for 5 min each. We dehydrated the samples in an increasing ethanol series (30 per cent → ethanol absolute), critical point dried them over CO_2_ (Balzers CPD 030) and coated them with 6 nm platinum (Quorum Q150T ES). A Zeiss Auriga Crossbeam was used for imaging.

### 2.2 DNA collection and sequencing strategy

We based our choice of which time points to sequence on the already available information about the dynamics of coevolutionary change in this experimental system. Previous work showed that the initial arms race between the two species changed to a fluctuating selection regime after a third round of resistance increase in the host populations, which was consistently observed at ∼Day 60 of the coevolution experiments (Frickel, Sieber, and Becks 2016; Retel et al. 2019a). Here we chose to focus on viral genomic variation during the arms race phase and sequenced populations sampled between Days 12 and 64 of the experiments (52 days of coevolution; the precise sampling scheme of experimental treatments and time points can be found in Supplementary Table S1). During this period, the coevolving host acted as an agent of directional selection on increased host clone range, and two rounds of host clone range increase occurred. Virus generation time is environment-dependent and therefore not easy to infer, but we estimated that 52 days of coevolution corresponded to approximately seventy-five generations of Chlorovirus evolution.

For DNA extraction, we took 40-ml samples from each chemostat. Ultracentrifugation was performed at ∼35,000 g for 2 h, before the pellet was frozen at −80**°**C. We then used DNeasy Blood and Tissue kits to extract DNA from this (chemostat community) sample, with minor modifications: First, 200 μl of concentrated sample was incubated at 56**°**C for 4 h together with 100 μl of buffer ATL and 30 μl of Proteinase. Afterward, we added 600 μl of 1:1 buffer ATL + ethanol mix and followed the standard column-based protocol.

We prepared sequencing libraries with Illumina NexteraXT kits. All libraries were 150-bp paired-end libraries. The three strong demography replicates, as well as the ancestral populations, were sequenced on four runs of an Illumina NextSeq machine (a subset of a previously published dataset; Retel et al. 2019a; Sequencing Read Archive bioproject PRJNA548271). The other three populations of the Weak Demography treatment were sequenced on two lanes of an Illumina NovaSeq S1.

### 2.3 Read quality control, alignment and variant calling

We used fastp v0.20.0 to assess sequencing quality and preprocess the raw sequencing data (Chen et al. 2018); we checked for Illumina adapter sequences, trimmed poly-g tails according to fastp’s default settings (—trim_poly_g), merged forward and reverse reads in case they overlapped (—merge —overlap_len_require 20 —overlap_diff_limit 5 —overlap_diff_percent_limit 5), pruned the right-end tail of a read when the average phred-scaled base calling quality dropped below 15 (—cut_right —cut_mean_quality 15) and removed reads shorter than 70 base calls (—length_required 70).

We aligned the reads with bwa mem v0.7.17 in paired-end mode (single-end mode for merged read pairs) (Li and Durbin 2009) to a reference genome downloaded from the National Center for Biotechnology Information (NCBI) Nucleotide database (NCBI reference sequence NC_000852.5) (Yanai-Balser et al. 2010; Agarwala et al. 2018). We checked mate information (samtools v1.9 fixmate) and added read groups (picard v2.0.1 AddOrReplaceReadgroups) before merging the paired- and single-end files with samtools merge (Li et al. 2009; http://broadinstitute.github.io/picard, last accessed 18 January 2022). We sorted (picard SortSam) and cleaned (picard CleanSam) the resulting .bam files, before calculating the depth of sequencing coverage (i.e. the number of reads covering a base in the reference genome) in 1 kb windows with samtools bedcov. For all but one population the mean coverage was above 1,000×; in this case we randomly sampled the aligned reads (samtools view -s) to reach a mean per-base sequencing coverage of 1,000×. The mean and median values per population and downsampling parameters can be found in Supplementary Table S1. Files were indexed using samtools (samtools index).

We used freebayes in pooled sequencing mode to find putatively polymorphic loci across the set of sequenced populations (—pooled-continuous -F 0.002 -C 1 —use-best-n-alleles 3). We required a sequencing coverage of at least 10× to include an allele frequency estimation. We implemented several filters to remove spurious variant calls caused by sequencing errors and artifacts. We started by excluding single nucleotide polymorphisms (SNPs) with a freebayes genotyping quality less than 20. Of the SNPs with a variant quality of at least 20, we implemented a missing data filter of 10 per cent (i.e. any variant with more than 3 NA values across the thirty-five sequenced populations was removed).

We further excluded putative variants from analysis if all pairs of alternative:reference allele counts per evolutionary replicate were draws from a single binomial distribution. Given that these Chlorovirus populations experienced strong directional selection on host clone range increase and that PBCV-1 reproduces clonally (so selection acting on any locus influences genomic variation across the whole genome), it is highly improbable for a polymorphic allele to remain at constant frequency over the time course of our experiments. Hence, a highly consistent allele frequency estimate is an indication that a putative variant is an artifact. In order to identify such artifacts, we took the number of alternative and reference observations per time point and added them up to calculate the overall proportion of alternative observations per replicate (per variant across time points). Then, for every empirical alternative:reference observation pair we calculated the probability that it was a draw from a binomial distribution with a success probability equal to the overall proportion of alternative observations (calculated per locus per replicate). If at any locus there was no False Discovery Rate (FDR)-corrected probability lower than 0.05, we concluded that there was no evidence that the allele in question had changed in frequency over time. In this case it is likely an artifact and we removed it from further analysis.

The last property we used to identify artifacts was the empirical rate of polymorphism. Spurious variation caused by for example complex genetic regions or duplication events is likely to result in consistently polymorphic alleles, and it is highly unlikely for any locus to be polymorphic across all replicates and time points. We removed any locus from analysis if its allele frequency estimate was ≤ 0.99 and > 0.01 across every population in a sequencing batch.

Of the 116 SNPs with a freebayes variant calling quality of 20 or higher, our criteria for removal based on NA values, based on the empirical observations being draws from a single binomial distribution and based on consistent polymorphism were met by, respectively, 6, 37 and 48 putative variants. The combination of these filtering steps resulted in a dataset of sixty-seven high-quality SNPs identified across six replicates (Supplementary Table S2).

### 2.4 Further genomic analysis

We visualized the genome-wide distribution of variation per population along the reference genome with the circlize package in R (R Core Team 2008; Gu et al. 2014). Based on the vectors of observed allele frequencies at the sixty-seven variable sites and an estimated number of 3 × 10^5^ sites reliably evaluated, we calculated expected heterozygosity as a measure of genetic diversity per population (Nei 1979). We used a *t*-test to test for statistical differences in genetic diversity at the end of the arms race phase of the experiment between the two treatments. We also ran a generalized linear model on the number of variable sites (the number of allele frequency estimates > 0) per population and treatment as explanatory variable, to test for differences in the number of polymorphic sites between treatments. Here we modeled the response as a Poisson distribution with a logarithmic link function.

To get a better idea of what types of mutations these PBCV-1 populations acquired, we used snpeff v4.3 m to classify the SNPs into intergenic, synonymous, non-synonymous and gained/lost start and stop codons (Cingolani et al. 2012). We then assessed if the empirical distribution of phenotypic effects conformed to the null expectation under a uniform per-position mutation probability. We did this by generating a .vcf file with every possible single nucleotide variant at every reference position and also running snpeff on this file. We ran Pearson’s Chi-squared test of independence to test for a statistical difference between the empirical and the null distribution. Because the total number of observed SNPs was relatively small, we generated a *P*-value by Monte Carlo sampling of test statistics under the null hypothesis (Hope 1968).

**Table 1.** Phenotypic and genomic statistics of the six experimental replicates. ‘Population’ identifies the experimental replicate; ‘Treatment’ describes the demographic treatment. Columns three and four report host clone range at the last measure time point and the average increase per day (values were obtained by a linear regression per replicate of the values shown in Fig. 1D, E). ‘Number of polymorphic sites’ corresponds to the number of alleles with a frequency > 0 at the last time point sequenced, and ‘nucleotide diversity’ is a measure of population genetic diversity.

Population	Treatment	Host clone range at last time point	Host clone range increase per day	Number of polymorphic sites	Nucleotide diversity
WD_1	Weak demography	0.806	0.0125	34	1.115 × 10^−5^
WD_2	Weak demography	0.806	0.0127	44	1.537 × 10^−5^
WD_3	Weak demography	0.954	0.0164	38	0.717 × 10^−5^
SD_1	Strong demography	0.933	0.0153	54	0.334 × 10^−5^
SD_2	Strong demography	0.889	0.0140	56	0.662 × 10^−5^
SD_3	Strong demography	0.878	0.0137	52	0.705 × 10^−5^

Following this, we investigated if non-synonymous versus synonymous mutations showed different patterns of repeatability and frequency change. As a measure of repeatability, we took the number of end point populations in which a mutation had a frequency larger than 0. We tested for correlations between predicted phenotypic effect and our measure of repeatability (number of replicates) with a Mann–Whitney test (Mann and Whitney 1947). As a measure of frequency change, we calculated the effective selection coefficient per SNP per replicate according to the formula provided in Kosheleva and Desai (2017). This effective selection coefficient gives the average additional progeny per generation of any individual possessing the concerning SNP, compared to a lineage whose frequency remains exactly constant. It here served primarily as a normalized measure of mean allele frequency change between Days 12 (start) and 58 or 64 (end point populations). We then calculated the average effective selection coefficient per SNP across the experimental replicates in which it was present. We tested for a correlation between predicted phenotypic effect and frequency change (mean effective selection coefficient) with a *t*-test. The presence of a significant difference between synonymous and non-synonymous SNPs of either repeatability or frequency change would indicate that different evolutionary forces operated on synonymous versus non-synonymous genetic variation.

Some of the acquired mutations were located in close proximity of each other. This observation prompted us to investigate if the observed genome-wide patterns of variation were concentrated in specific genes. For every one of the 812 open reading frames (ORFs) in the PBCV-1 reference genome, we made a contingency table with total genome length, length of the ORF, the genome-wide number of sites at which a mutation was observed, and the number of mutated sites in the specific ORF. We performed Fisher’s exact test per contingency table and corrected the set of resulting *P*-values for family-wise error rate using Holm’s method (Holm 1979). This approach told us which genes (if any) acquired more mutations during the experiments than expected under a uniform genome-wide probability, corrected for the length of the gene.

Finally, we compiled the available functional information of the ORFs in which SNPs were found. For all ORFs with at least one variable site, we assembled information on gene expression profiles (Yanai-Balser et al. 2010; Blanc et al. 2014), gene conservation among the family of *Phycodnaviridae* (Jeanniard et al. 2013; Seitzer et al. 2018) and several proteomic characteristics of the encoded proteins (Dunigan et al. 2012; Fang et al. 2019), and we performed protein–protein BLAST queries (Altschul et al. 1997). The goal of this functional annotation was to characterize the genomic basis of increased host clone range in Chloroviruses and to elucidate how the two species might interact on the molecular level.

## 3. Results

### 3.1 Population size and phenotypic change

The temporal dynamics of census population size and host clone range are shown in Fig. 1B–E. The minimum observed census population size across six replicates was 3.2 × 10^9^, with harmonic means across 60 days of 7.5 ± 4.0 × 10^10^ (mean ± SD across six replicates) (Fig. 1B, C). Because of these large census population sizes, we anticipated that the differences between the two treatments (weak and strong demography) might not impact the phenotypic and molecular evolution of the evolving PBCV-1 populations much. Indeed, phenotypic change was characterized by increases in host clone range between Days ∼20 and 60 in both experimental treatments (Fig. 1D, E) and an overall remarkable repeatability. We found no statistical evidence for a between-treatment difference in the host clone range over time (Table 1 and linear regression model; *P* = 0.063).

### 3.2 Distribution and repeatability of genomic changes

**Figure 2.**
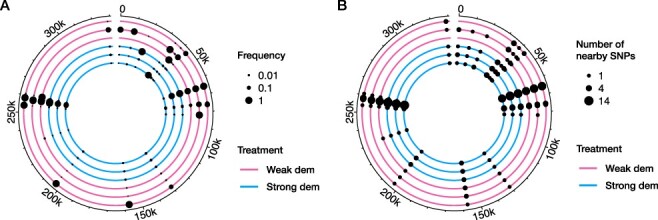
Genomic change in experimentally evolved Chlorovirus populations. Every colored line reflects the genome of one out of six replicate populations, with the ones that evolved under reduced population size fluctuations (weak demography) colored pink and those that evolved under strong population size fluctuations (strong demography) blue. Reference genome position runs clockwise starting from the top. In (A), every dot corresponds to a *de novo* SNP, with the size of the dot reflecting the frequency in the population. Several groups of SNPs were found in very close proximity to each other, making the lower-frequency ones undetectable in this figure. To resolve this, we plotted the same set of SNPs in (B), but with the size of the dots corresponding to the number of variants found within 500 bp, including the focal variant.

Across the six analyzed experimental replicates, we identified sixty-seven unique SNPs (Fig. 2) with 30 ± 11.5 (mean ± SD across six replicates) per replicate. Only two of the observed SNPs were found in only one replicate while most observed SNPs were present in multiple replicates (on average in 4.1 replicates). The number of variable sites was consistently higher in the populations in the strong demographic versus the weak demographic treatment (log-linear regression of the number of variable sites at the last time point over treatment; *P* = 0.006). There was no difference in genome-wide diversity between the two treatments (*t*-test comparing nucleotide diversities at the last time point between treatments, *P* = 0.13).

**Figure 3.**
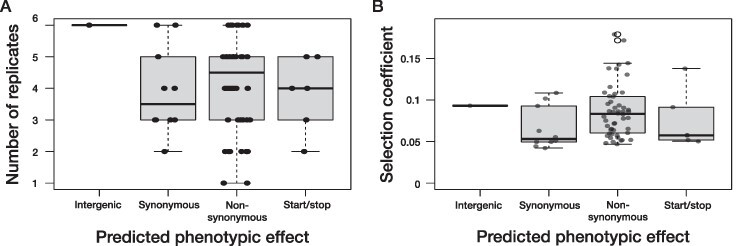
The relationship between predicted phenotypic effect and the magnitude and repeatability of genomic change. A) For every one of the sixty-seven observed SNPs, the number of replicates in which it was found is shown on the vertical axis, with the corresponding predicted phenotypic effect on the horizontal axis. There was no statistical difference between the number of replicates with non-synonymous SNPs versus synonymous SNPs (Mann–Whitney *U* test, *P* = 0.466). B) The effective selection coefficient (a measure of relative increased growth per generation compared to a lineage that remains constant in frequency) was calculated per SNP and averaged across replicates and is here plotted against its predicted phenotypic effect on the horizontal axis. Effective selection coefficients were higher for non-synonymous than for synonymous SNPs (*t*-test, *P* = 0.038). We did not include intergenic and start/stop changes in these statistics, because the predictions on their average fitness effects are less clear. Horizontal jitter was added to both figures.

**Figure 4.**
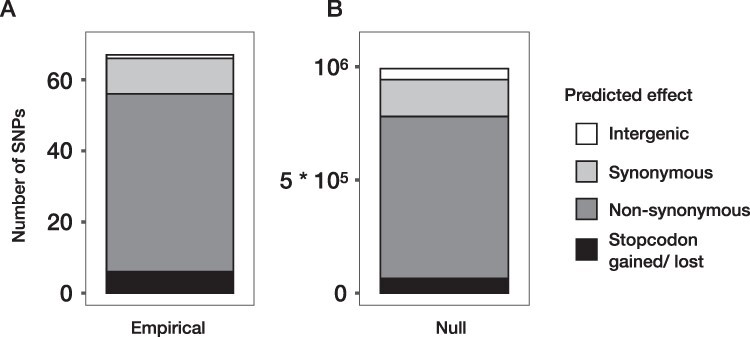
The distribution of predicted phenotypic effects of genomic changes observed during replicated experimental evolution of Chlorovirus populations, obtained using snpeff (Cingolani et al. 2012). (A) The empirical distribution, i.e. the predicted phenotypic effects of the sixty-seven SNPs detected in the studied populations. (B) The genome-wide null distribution, i.e. the set of predicted phenotypic effects of all possible base changes at every position of the reference genome.

We ran snpeff to determine the predicted phenotypic effects of the set of observed SNPs. One of the 67 SNPs was located in an intergenic region, which covers ∼5 per cent of the total genome. Start and stop codons were gained or lost six times. Between synonymous and non-synonymous SNPs, we found no statistical evidence for difference in the number of replicates a variant was present (Fig. 3A and Mann–Whitney *U* test, *P* = 0.466). Average frequency change was on the other hand higher for non-synonymous than for synonymous SNPs (Fig. 3B and *t*-test comparing effective selection coefficients between the two classes, *P* = 0.038). The empirical (Fig. 4A) and null distributions (Fig. 4B) were not significantly different (Pearson’s Chi-squared test of independence, *P*-value = 0.50).

**Figure 5.**
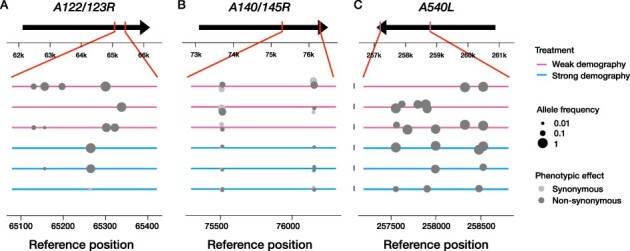
Genomic variation in the three genes with excess variable sites. The arrows show the three ORFs *A122/123R* (A), *A140/145R* (B) and *A540L* (C), which had significantly more variable sites than expected under a uniform probability of polymorphism. Reference genome position is given on the horizontal axis, and the direction of the arrow shows the direction of transcription. The six colored lines represent the six replicate Chlorovirus populations, with the ones that have undergone strong population size fluctuations in blue and those that have not in pink. Every dot corresponds to a SNP, is colored by its predicted phenotypic effect and has a size corresponding to its estimated allele frequency. Vertical jitter was added to figures (B) and (C) to increase visibility of neighboring SNPs.

Mutations were not uniformly distributed across the genome, but they were often located in close proximity to each other, with clusters of SNPs found at up to fourteen neighboring sites (Fig. 2B). Because the allele frequencies of neighboring SNPs within a population were often dissimilar (Fig. 5), we knew that they were caused by independent mutation events (and they were not multi-nucleotide polymorphisms inadvertently classified as multiple SNPs). To test if there were any genes that acquired more mutations than expected by chance, we performed Fisher’s exact test for every putative ORF of the PBCV-1 reference genome. This revealed that three genes *A122/123R, A140/145R* and *A540L* had significantly more variable sites than expected under a uniform mutation probability across the reference genome (Fisher’s exact test, largest Family-Wise Error Rate (FWER)-corrected *P*-value 8.2 × 10^−11^). We zoomed in on those three genes and visualized the genetic variation in Fig. 5. All but one of the SNPs in A122/123R and A540L were non-synonymous. The SNPs in *A140/145R* were a mix of non-synonymous and synonymous changes (four non-synonymous versus three synonymous SNPs). Especially in genes *A122/123R* and *A140/145R*, all variations occurred in a confined region within the gene.

**Figure 6.**
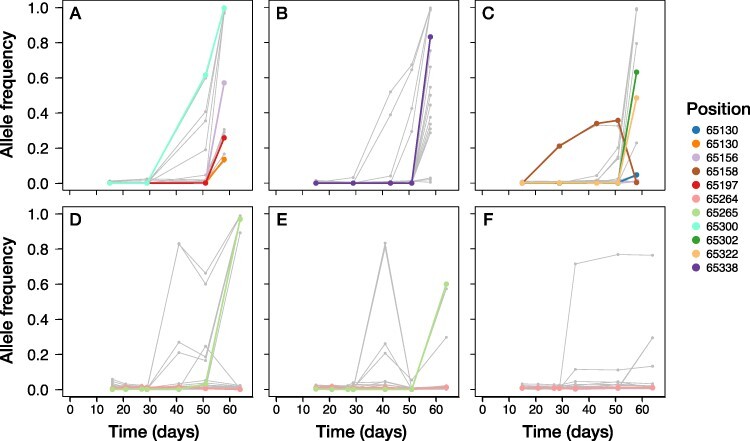
Temporal dynamics of mutations in ORF *A122/123R*. The allele frequency of every SNP detected over 60 days of experimental evolution is represented by a gray line. All SNPs located in *A122R* (one of the three genes with excess variable sites) that reached a frequency of at least 1 per cent in the specific replicate are colored. When we observed two different base changes at the same position in different experimental replicates, we classified them as separate polymorphisms and gave them different colors. The top three panels (A–C) correspond to the experimental replicates with weak population size fluctuations, and the bottom panels (D–F) correspond to replicates with strong population size fluctuations.

**Figure 7.**
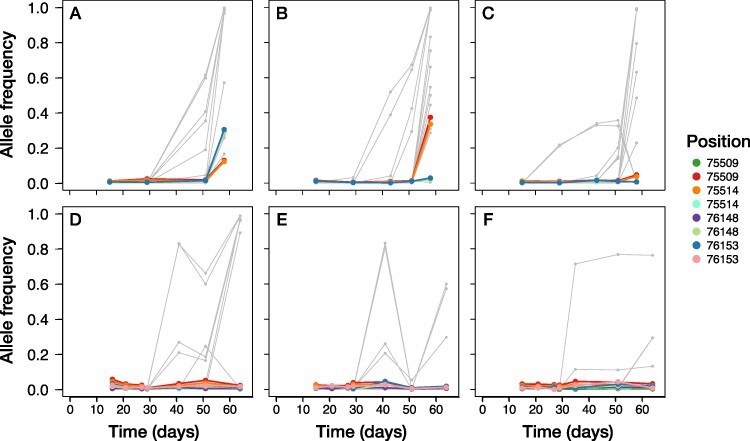
Temporal dynamics of mutations in ORF *A140/145R*. The allele frequency of every SNP detected over 60 days of experimental evolution is represented by a gray line. All SNPs located in *A140/145R* (one of the three genes with excess variable sites) that reached a frequency of at least 1 per cent in the specific replicate are colored. When we observed two different base changes at the same position in different experimental replicates, we classified them as separate polymorphisms and gave them different colors. The top three panels (A–C) correspond to the experimental replicates with weak population size fluctuations, and the bottom panels (D–F) correspond to replicates with strong population size fluctuations.

**Figure 8.**
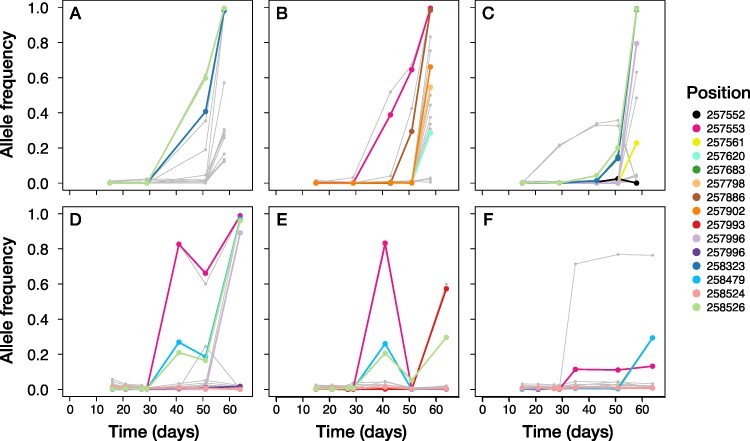
Temporal dynamics of mutations in ORF *A540L*. The allele frequency of every SNP detected over 60 days of experimental evolution is represented by a gray line. All SNPs located in *A540L* (one of the three genes with excess variable sites) that reached a frequency of at least 1 per cent in the specific replicate are colored. When we observed two different base changes at the same position in different experimental replicates, we classified them as separate polymorphisms and gave them different colors. The top three panels (A–C) correspond to the experimental replicates with weak population size fluctuations, and the bottom panels (D–F) correspond to replicates with strong population size fluctuations.

The temporal dynamics of genomic change in these three genes differed (Figs 6–8). All of the variants in *A122/123R* (except for one SNP at Position 65,158 in replicate WD_3; Fig. 6C) reached a detectable frequency only at the very last time point. In contrast, frequency increases of the mutations in *A540L* were usually (five out of six replicates) first observed in the time interval following Day 30 (Fig. 8A–F). The SNP at Position 258,526 has a highly correlated allele frequency with either one at Position 258,323 or 258,479. The derived mutations in ORF *A140/145R* remained minor alleles in all six experimental replicates, reaching a maximum frequency of 30.5 per cent in replicate WD_1 (non-synonymous SNP at Position 76,153; Fig. 7A) and 37.4 per cent in WD_2 (synonymous SNP at Position 75,509; Fig. 7B). In the other four experimental replicates (WD_3 and the three replicates including strong demography; Fig. 7C–F), the estimated allele frequencies of all SNPs in this ORF remained below 6 per cent.

### 3.3 Molecular functions of the genes in which mutations occurred

SNPs were found in eighteen ORFs. Here we describe the available information on their molecular function, gene expression profiles, degree of conservation and the results of our protein–protein BLAST queries, starting with the three genes with excess variable sites. This information is also summarized in Table 2.

**Table 2.** Functional annotations of genes in which we found mutations. The first four columns give the name of the ORF (‘Name’), genomic coordinates (‘Start’ and ‘End’) and length of the gene. Columns five until nine correspond to information extracted from other studies, respectively whether the protein product is present in the virion, its relative abundance if it is, gene expression patterns according to two different studies and the gene gang the ORF is a part of. Column ten gives the number of unique SNPs we found among six evolutionary replicates (‘No. of variable sites’), and column eleven gives the number of unique SNPs times the number of independent replicates they were found (‘No. of SNPs found’. Column ‘FET P-value’ gives the P-value of Fisher’s exact test carried out to assess if an ORF had an excess number of variable sites. Relevant results of our protein–protein BLAST queries are summarized in the second to last column (‘BLAST results’), and we give references mentioning or investigating the specific ORF in the last column.

Name (PBCV-1 gene tag)	Start	End	Length	Virion-associated (Dunigan et al. 2012)	Relative abundance in virion proteome (Dunigan et al. 2012)	Gene expression microarray (Yanai-Balser et al. 2010)	Gene expression RNAseq (Blanc et al. 2014)	Gene gang (Seitzer et al. 2018)	No. of variable sites	No. of SNPs found	FET *P*-value	BLAST results	Additional citations
a001L	280	549	270	No		NA	NA		2	2	1	No hits	
A014R	8255	12,364	4110	No		Late	2.2		1	3	1	No hits	
A025/027/029L	16,432	20,511	4080	Yes	0.07	Late	2.2		2	2	1	Glycoprotein-repeat domain containing protein	
A064R	34,956	36,872	1917	No		Early	2.1		3	7	1	Glycosyltransferase domains; involved in glycosylation of Vp54	(Graves et al. 2001; De Castro et al. 2013, 2018)
A075bL	39,078	39,920	843	No		Early-Late	2.2		2	6	1	Exostosin gene	
A078R	42,007	42,183	177	No		Early	1.2		2	5	0.520	No hits	
A081L	43,192	43,761	570	No		Early	2.2		2	3	1	Only hits are other Chloroviruses	
A122/123R	62,145	66,176	4032	Yes	NA	Late	2.2		14	32	8.1 × 10^−11^	Encodes glycoprotein Vp260	(Chuchird et al. 2002; Zhang et al. 2011)
A140/145R	73,107	76,490	3384	Yes	3	Early-Late	2.1	6	7	30	0.005	Encodes host recognition protein Vp130	(Onimatsu et al. 2004; Onimatsu et al. 2006)
A162L	81,717	82,952	1236	No		Early	1.3		1	4	1	Glutamate receptor	
A256/257L	129,608	132,121	2514	Yes	0.03	Early-Late	1.2		1	5	1	No hits	
A314R	157,306	157,548	243	Yes	0.38	Late	2.2		2	8	0.966	Only hits are other Chloroviruses	
A416R	202,802	203,368	567	No		Late	2.2	11	2	5	1	Kinase protein	
A478L	230,676	231,608	933	No		Early-Late	2.1		1	4	1	Zn-finger containing protein	
A533R	253,765	254,889	1125	Yes	0.35	Early-Late	2.1	1	2	5	1	Only hits are other Chloroviruses	
A537L	255,643	256,440	798	No		Early	1.1		1	5	1	Only hits are other Chloroviruses	
A540L	257,089	260,859	3771	Yes	0.17	Late	2.2	1	20	50	5.0 × 10^−20^	Glycoprotein-repeat domain containing protein	
A689L	328,590	329,015	426	No		NA	2.2		2	3	1	No other hits	

ORF *A122/123R* is one of the three ‘variation hotspots’, with SNPs occurring at fourteen different sites. *A122/123R* is the gene that encodes glycoprotein Vp260 in PBCV-1, which is one of the minor capsid proteins that make up the outer envelope of the virus particle. It therefore plays an important role in the structural integrity and solubility of the virion (Zhang et al. 2011).

The second variation hotspot was ORF *A140/145R,* where we observed SNPs at seven different sites during our experiments. *A140/145R* (also referred to as *Vp130ORF*) encodes for protein Vp130, which is the spike-like protein that recognizes and binds to the outer host cell membrane (Onimatsu et al. 2004, 2006).

The third ORF with an excess number of variable sites was *A540L*. This gene is unique to Chloroviruses but highly conserved within the family (Jeanniard et al. 2013) and part of a ‘gene gang’ (Seitzer et al. 2018). A gene gang is a syntenic cluster of genes that colocalizes across Chlorovirus strains, often with a shared cellular function (e.g. DNA replication). Gene gangs are somewhat equivalent to bacterial operons but without the coordinated mRNA transcription patterns. The functionality of this particular gang is not known. *A540L* is expressed in the late stage of infection and its protein product is virion-associated. Protein–protein BLAST queries revealed that the protein contains a glycoprotein-repeat domain.

Besides the three genes with excess variable sites, we found three polymorphic sites in ORF *A064R*. This ORF is highly conserved within the family of Chloroviruses (Jeanniard et al. 2013). It has a glycosyltransferase domain and is involved in the synthesis of the major capsid protein Vp54 (Graves et al. 2001), which makes up most of the virion envelope. Chloroviruses with truncated versions of *A064R* are viable but less stable (De Castro et al. 2013, 2018).

Of the total eighteen ORFs with variable sites, five (*a001L, A014R, A078R, A256*/257L and *A689L*) showed no homology to any protein in the NCBI database. Five other ORFs (*A025/027/029L, A081L, A314R, A533R* and *A537L*) only produced hits to (putative) proteins in other Chloroviruses. *A533R* is part of the same gene gang as *A540L* mentioned above (Seitzer et al. 2018). The remaining SNPs were found in ORFs *A075L* (significant homology to the human Exostosin gene), *A162L* (which has a putative glutamate receptor domain), *A416R* (which shows homology to kinases in other viruses and some bacteria) and *A478L* (which has a putative Zn-finger domain). Of these four ORFs, the homologous proteins in other organisms are broadly involved in metabolism and/or signal transduction.

## 4. Discussion

Here we investigated the genomic change observed in Chlorovirus PBCV-1 populations during coevolution with their host under controlled experimental conditions. Our goals were to investigate the forces influencing molecular evolution, which is not yet well understood for Chloroviruses, as well as to identify genes putatively involved in the coevolutionary arms race with *C. variabilis*.

The repeatability of genomic change was pervasive, with all but two SNPs found in more than one evolutionary replicate (Figs 2 and 3A). We interpret this as evidence that positive selection was an important force shaping genome-wide variation in these Chlorovirus populations. The patterns of repeatability observed here fit in with a range of investigations into viral genomic change, coming both from clinical (e.g. Bacheler et al. 2000; Subramanian, Vijayalingam, and Chinnadurai 2006; Koel et al. 2013) and experimental evolution investigations (Wichman et al. 1999; Paterson et al. 2010; Scanlan et al. 2011; Marston et al. 2012; Perry, Barrick, and Bohannan 2015). Non-synonymous SNPs on average also reached higher frequencies than synonymous SNPs (Fig. 3B), which is another indication of positive selection (McDonald and Kreitman 1991; Nielsen and Yang 1998; Hahn, Rausher, and Cunningham 2002).

More than half of the observed SNPs were found in at least five out of six evolutionary replicates. This means that mutation events occurred independently at these sites in most replicates, which in turn indicates that almost every possible base change at every position of the genome was likely to successfully establish within the timeframe of 50 days or approximately seventy-five generations. Based on this we conclude that these PBCV-1 populations were not mutation-limited. This was not a surprise given that census population sizes remained relatively high: Multiplying the harmonic mean population size with the lowest estimate of mutation rate reported for dsDNA viruses (2 × 10^−8^ base changes per nucleotide per replication event; Sanjuán et al. 2010) suggests that ∼1,500 virus particles might acquire a *de novo* SNP at a specific reference position every generation. However, a lot of uncertainties still exist about the relationship between the number of particles in a viral population and the rate by which it generates new genetic variation (Duffy, Shackelton, and Holmes 2008; Pennings, Kryazhimskiy, and Wakeley 2014; Eldon 2020). That the Chlorovirus populations in our flasks appeared not mutation-limited implies that waiting time for new mutations might generally not be long for dsDNA virus populations in larger lakes. Ample *de novo* mutation as the primary source of genetic variation is consistent with the observation that a lot of PBCV-1 genes have no functional orthologs outside the family of Chloroviruses (Jeanniard et al. 2013). The lack of mutation limitation also provides an interesting contrast to observations for some human pathogens: for example, HIV reaches similarly high population sizes within a single human host, but *de novo* mutation is still limiting (Pennings, Kryazhimskiy, and Wakeley 2014).

The six experimental replicates were separated into two treatments involving either weak or strong population size fluctuations, corresponding to a weak or strong demographic influence on genomic variation. The theoretical expectation is a positive correlation between (effective) population size and the fixation rate of adaptive mutations (hence the speed of adaptation), but this correlation breaks down when mutations are no longer limiting (De Visser and Rozen 2005). Our observations of a high degree of genomic repeatability across experimental replicates and no differences in patterns of phenotypic (host clone range) and genomic variation of PBCV-1 between the two experimental treatments both correspond well with the theoretical expectation. We also found no evidence for differences in the rate of phenotypic evolution (changes in host clone range) and in population genetic diversity at the end of the experiments. However, three replicates per treatment might limit our ability to detect settled differences between the two demographic treatments. That we consistently observed higher numbers of polymorphic sites in the populations evolving under strong demography might indicate that. Overall, our results suggest that the viral populations were not limited by mutations in either of our treatments.

Synonymous and non-synonymous SNPs were equally likely to occur (Figs 3A and 4). This was somewhat puzzling, considering that high repeatability (Figs 2 and 3A) and higher non-synonymous allele frequencies (Fig. 3B) led us to the conclusion that positive selection shaped genome-wide variation. One potential explanation is that a lot of the synonymous mutations we observed were beneficial, which fits in with a growing awareness of the non-neutrality of synonymous mutations (Cuevas, Domingo-Calap, and Sanjuán 2012; Fredens et al. 2019; Lebeuf-Taylor et al. 2019). We can however not rule out that the limited overall number of variable sites led to a lack of power to detect differences in occurrence probability between synonymous and non-synonymous SNPs. To investigate to what extent synonymous and non-synonymous variation is governed by different forces, one would ideally investigate their patterns of genetic diversity separately (Hahn, Rausher, and Cunningham 2002; Nielsen and Slatkin 2013). However, in our case such a separation resulted in very sparsely sampled site frequency spectra, which is why we refrained from running any separate analysis on them. Overall, the patterns of genomic variation in our dataset were entirely consistent with prevalent positive selection acting on synonymous as well as non-synonymous SNPs.

We found three genes with excess variables sites (‘variation hotspots’) *A122R, A140/145R* and *A540L* (Fig. 5). The large number of mutations and high repeatability of genomic change observed in these genes suggested that they were especially important during coevolution with the host. *A122R* encodes for one of the minor capsid proteins that make up the outer envelope of the virus particle. The mutations in *A122R* (fourteen polymorphic sites, all non-synonymous except one) only increased in frequency at the very last time point sequenced in all six evolutionary replicates (Fig. 6). This suggests that changes to the virion capsid became adaptive later in the experiments, after the first rounds of resistance and host clone range increase. Different strains of Chloroviruses encode for a variable number of minor capsid proteins, and they are hypothesized to have (partially) overlapping functionality (in the sense that viable virus particles can be created with multiple combinations of relative amounts of capsid proteins) (Chuchird et al. 2002). PBCV-1 is special in the sense that it synthesizes only two minor capsid proteins, making it likely that the genes encoding these are rather essential to complete a life cycle.

The second hotspot *A140/145R* harbored a mixture of synonymous and non-synonymous mutations. This gene encodes for the spike-like protein on the outside of every PBCV-1 virion, used for host cell recognition, attachment and initial entry. Resistance to PBCV-1 seems by and large a binary property of the host individual (Frickel, Sieber, and Becks 2016). Therefore, we hypothesized that recognition of the cell wall was important in determining whether or not an infection event was successful. Repeated non-synonymous changes in Vp130 fit our idea that changes in the way the virus recognizes its host (and in how the host prevents recognition) drive the arms race between the two species (Frickel, Sieber, and Becks 2016). However, the temporal dynamics revealed that the SNPs in this gene in most replicates never reached a frequency that could explain a population-wide phenotypic change (Fig. 7). This makes it unlikely that the elevated levels of polymorphism in this specific gene were driven by selection on increased host clone range alone. An alternative explanation for the persistence of polymorphism throughout the experiments in this gene might be a locally increased *de novo* mutation rate. This was recently proposed as a mechanism for adaptation in another microbial coevolutionary system, where continuous *de novo* mutation in a large population effectively resulted in a pool of standing genetic variations, some of which were adaptive (Gupta et al. 2020). A significantly increased number of polymorphic sites, a mixture of non-synonymous and synonymous changes and the persistence of low-frequency variation throughout our experiments are all consistent with a putatively elevated mutation rate in *A140/145R*, the protein that encodes PBCV-1’s spike-like host recognition protein.

In the third gene with excess variable sites *A540L*, frequency increases of mutations often occurred in the time interval following Day 30. This coincided with the first increase in host clone range (Fig. 1) and suggests a role for A540L in PBCV-1’s ability to infect its algal host. *A540L* is highly conserved in the family of Chloroviruses and part of a gene gang. We also found a SNP in *A533R*, another member of the same gene gang. All of the protein products of this gang are virion-associated, but most of them only show homology to other putative Chlorovirus proteins, and their expression patterns during infection did not show a consistent pattern (Seitzer et al. 2018). All of this information suggests that *A540L* is essential for PBCV-1, but its precise molecular function is as of yet unknown.

In conclusion, our investigation showed that experimentally evolving Chlorovirus PBCV-1 populations experienced strong positive selection and were not mutation-limited. Such high prevalence of spontaneous mutations over short timescales is not generally observed for viruses, despite their large population sizes, short generation times and high mutation rates. The high mutation prevalence helped us find several ‘variation hotspots’ and hence identify putative targets of selection during the coevolutionary arms race between PBCV-1 and its host *C. variabilis* NC64A. Although population size and phenotypic dynamics were similar between replicates, and the same variants were often found in multiple experimental replicates, patterns of allele frequency change over time of these variants were less repeatable. By highlighting the important evolutionary processes that shaped the dynamics of genomic change in a common strain of freshwater algal viruses, we increased our understanding of how viruses evolve. Our investigation is a step forward to elucidate the rapid adaptation of large dsDNA viruses in natural aquatic ecosystems.

## Supplementary Material

veac003_Supp

## Data Availability

We uploaded the sequencing data for this study to the European Nucleotide Archive at European Molecular Biology Laboratory - European Bioinformatics Institute under accession number PRJEB46915 (https://www.ebi.ac.uk/ena/browser/view/PRJEB46915). The population size and phenotypic data are available at opendata.eawag.org/ (https://doi.org/10.25678/0005BQ). Custom scripts mentioned in Materials and Methods are available at github.com/RetelC/ChlorovirusGenomicChange. Additional data may be requested from the authors.
