# Supplementary material for: Strong selection and high mutation supply characterize experimental Chlorovirus evolution
Source: Virus Evol. 2022 Jan 25;8(1):veac003. doi: 10.1093/ve/veac003 (PMC8838748; doi:10.1093/ve/veac003)
Supplement: veac003_Supp [file veac003_supp.zip › Retel_etal_Supplementary_data.docx]

Supplementary data

for

## Strong selection and high mutation supply characterize experimental *Chlorovirus* evolution

Cas Retel^1,2^, Vienna Kowallik^3,4^, Lutz Becks^3,5,6,*^, Philine G.D. Feulner^1,2,7,*^,

^1^Department of Fish Ecology and Evolution, Center for Ecology, Evolution and Bio-geochemistry, EAWAG, Swiss Federal Institute of Aquatic Science and Technology, Kastanienbaum, Switzerland.

^2^Division of Aquatic Ecology, Institute of Ecology and Evolution, University of Bern, Bern, Switzerland.

^3^Community Dynamics Group, Department of Evolutionary Ecology, Max Planck Institute for Evolutionary Biology, Plön, Germany.

^4^Current address: Ecology and Evolution Unit, Okinawa Institute of Science and Technology (OIST), Okinawa, Japan.

^5^Aquatic Ecology and Evolution, Limnological Institute University Konstanz, Konstanz, Germany.

^6^E-mail: lutz.becks@uni-konstanz.de

^7^E-mail: philine.feulner@eawag.ch

*These authors contributed equally to this work

Supplementary table 1. Depth of sequencing coverage per sequenced population. Column “Replicate” gives the experimental replicate (SD = Strong Demography, WD = Weak Demography), “Day” the time point when the population was sampled. “Breadth” corresponds to the fraction of 1Kb-windows of the reference genome covered by at least 1000 base pairs of high-quality aligned reads. The fourth and fifth columns show the Median and Mean values of the set of genome-wide sequencing coverages calculated per 1Kb window. The last column “Downsampling seed” is the input argument used for subsampling reads with samtools view -s (Li et al., 2009). The integer before the dot is a random seed, the fraction after the dot gives the fraction of total reads sampled.

| **Replicate** | **Day** | **Breadth** | **Median** | **Mean** | **Downsampling seed** |
| --- | --- | --- | --- | --- | --- |
| SD_1 | 16 | 0.996979 | 24935.8 | 25272.9 | 118.0395681 |
| SD_1 | 21 | 0.996979 | 10281.4 | 10372.5 | 812.0964088 |
| SD_1 | 27 | 0.996979 | 14382.2 | 14340.3 | 674.0697335 |
| SD_1 | 29 | 0.996979 | 7366.35 | 7415.78 | 491.1348476 |
| SD_1 | 41 | 0.996979 | 6714.67 | 6850.57 | 371.1459733 |
| SD_1 | 51 | 0.996979 | 19626.3 | 20066.9 | 903.0498333 |
| SD_1 | 64 | 0.996979 | 5009.05 | 5046.85 | 779.1981434 |
| SD_2 | 15 | 0.996979 | 7434.92 | 7453.39 | 944.1341671 |
| SD_2 | 21 | 0.996979 | 9745.56 | 9857.35 | 727.1014471 |
| SD_2 | 27 | 0.996979 | 10343.7 | 10436 | 802.0958222 |
| SD_2 | 29 | 0.996979 | 8942.91 | 9192.31 | 197.1087866 |
| SD_2 | 41 | 0.996979 | 9656.26 | 9870.56 | 668.1013114 |
| SD_2 | 51 | 0.996979 | 52962.5 | 51504.6 | 808.0194157 |
| SD_2 | 64 | 0.996979 | 5537.36 | 5638.44 | 169.177354 |
| SD_3 | 15 | 0.996979 | 8995.39 | 9028.73 | 639.1107575 |
| SD_3 | 21 | 0.996979 | 22506.4 | 23065.6 | 552.0433546 |
| SD_3 | 27 | 0.996979 | 18675.6 | 19160.2 | 782.0521915 |
| SD_3 | 29 | 0.996979 | 52530.6 | 50974.5 | 631.0196177 |
| SD_3 | 35 | 0.996979 | 16929.9 | 17353.3 | 445.0576259 |
| SD_3 | 51 | 0.996979 | 26685.8 | 27092.4 | 309.0369107 |
| SD_3 | 64 | 0.993958 | 628.336 | 634.607 | NA |
| WD_1 | 15 | 0.996979 | 47365.4 | 45798.9 | 402.0218346 |
| WD_1 | 29 | 0.996979 | 50472.7 | 48576.5 | 864.0205861 |
| WD_1 | 43 | 0.0120846 | 0.186 | 0.238511 | NA |
| WD_1 | 51 | 0.996979 | 11015 | 11305.4 | 421.0884533 |
| WD_1 | 58 | 0.996979 | 11749.6 | 11643.5 | 448.0858848 |
| WD_2 | 15 | 0.996979 | 27848.1 | 28267.1 | 569.0353768 |
| WD_2 | 29 | 0.996979 | 23293.5 | 23427.1 | 585.0426856 |
| WD_2 | 43 | 0.996979 | 7925.74 | 8370.09 | 360.119473 |
| WD_2 | 51 | 0.996979 | 9642.05 | 9951.04 | 652.100492 |
| WD_2 | 58 | 0.996979 | 2843.1 | 2841.46 | 729.3519318 |
| WD_3 | 15 | 0.996979 | 49594.6 | 47804.3 | 716.0209186 |
| WD_3 | 29 | 0.996979 | 43751.7 | 43003.6 | 514.0232539 |
| WD_3 | 43 | 0.996979 | 6188.9 | 6341.95 | 390.1576802 |
| WD_3 | 51 | 0.996979 | 14094 | 14646.8 | 117.0682743 |
| WD_3 | 58 | 0.996979 | 8339.1 | 7978.46 | 628.1253375 |

**Supplementary table 2**. The number of putative SNPs removed by our variant filtering steps. We carried out every filtering step on the ‘complete’ set of putative SNPs with a freebayes QUAL ≥ 20 (n = 116). Therefore, the figures in the right column reflect the number of SNPs out of those 116 that matched the corresponding criterium. Because some putative variants matched more than one criterium for removal, the numbers of the bottom four rows do not add up to 116.

| **Description** | **Number** |
| --- | --- |
| SNPs with a freebayes QUAL ≥ 20 | 116 |
| Removed because more than 10% missing data | 6 |
| Removed because observations were draws from a single binomial distribution | 37 |
| Removed because variants were consistently polymorphic | 48 |
| SNPs included in further analysis | 67 |
